# Supplementary material for: The deployment of ProKnow for cloud-based clinical research in radiotherapy
Source: PLOS Digit Health. 2026 Jul 17;5(7):e0001131. doi: 10.1371/journal.pdig.0001131 (PMC13378969; doi:10.1371/journal.pdig.0001131)
Supplement: S1 Table — (DOCX) [file pdig.0001131.s003.docx]

|  | Standard Plan (60 Gy) | | | Experimental Plan (75 Gy) | | |
| --- | --- | --- | --- | --- | --- | --- |
| Patient Number | EUD (Monaco) | EUD (ProKnow) | Difference (%) | EUD (Monaco) | EUD (ProKnow) | Difference (%) |
| Patient_01 | 49.4 | 49.5 | 0.2 | 54.0 | 54.0 | 0.0 |
| Patient_02 | 45.1 | 45.1 | 0.0 | 47.6 | 47.6 | 0.0 |
| Patient_03 | 50.5 | 50.6 | 0.2 | 56.4 | 56.4 | 0.0 |
| Patient_04 | 45.2 | 45.2 | 0.0 | 48.3 | 48.4 | 0.2 |
| Patient_05 | 50.4 | 50.4 | 0.0 | 54.7 | 54.7 | 0.0 |
| Patient_06 | 49.1 | 49.1 | 0.0 | 53.7 | 53.6 | 0.2 |
| Patient_07 | 49.0 | 49.0 | 0.0 | 54.3 | 54.3 | 0.0 |
| Patient_08 | 50.6 | 50.6 | 0.0 | 55.5 | 55.6 | 0.2 |
| Patient_09 | 49.3 | 49.4 | 0.2 | 53.3 | 53.5 | 0.4 |
| Patient_10 | 49.5 | 49.5 | 0.0 | 53.2 | 53.3 | 0.2 |
