# Supplementary material for: The deployment of ProKnow for cloud-based clinical research in radiotherapy
Source: PLOS Digit Health. 2026 Jul 17;5(7):e0001131. doi: 10.1371/journal.pdig.0001131 (PMC13378969; doi:10.1371/journal.pdig.0001131)
Supplement: S2 Table — (DOCX) [file pdig.0001131.s004.docx]

| **STRUCTURES** | **DICE COEFFICIENTS: "03-XXX-DUMMY"** | | | |
| --- | --- | --- | --- | --- |
| **ROI Name** | **GOLDEN RULE** | **PLASTIMATCH** | **DIFFERENCE (%)** | |
| **Brain** | 0.948 | 0.948 | 0.0 | |
| **Brain-GTV** | 0.944 | 0.944 | 0.0 | |
| **Brainstem** | 0.803 | 0.803 | 0.0 | |
| **Brainstem_Centre** | 0.797 | 0.793 | -0.5 | |
| **Brainstem_PRV** | 0.809 | 0.810 | 0.1 | |
| **Cochlea_L** | 0.657 | 0.619 | -6.0 | |
| **Cochlea_R** | 0.7 | 0.698 | -0.3 | |
| **CTV60** | 0.905 | 0.905 | 0.0 | |
| **CTV60ex** | 0.896 | 0.896 | 0.0 | |
| **Eye_L** | 0.889 | 0.885 | -0.5 | |
| **Eye_R** | 0.858 | 0.856 | -0.2 | |
| **Glnd_Lacrimal_L** | 0.652 | 0.642 | -1.5 | |
| **Glnd_Lacrimal_R** | 0.545 | 0.547 | 0.4 | |
| **GTV** | 0.872 | 0.873 | 0.1 | |
| **GTVu** | 0.887 | 0.887 | 0.0 | |
| **Lens_R** | 0.764 | 0.767 | 0.4 | |
| **OpticChiasm** | 0.756 | 0.723 | -4.5 | |
| **OpticChiasm_PRV** | 0.813 | 0.816 | 0.4 | |
| **Optic_Nrv_L** | 0.796 | 0.783 | -1.6 | |
| **Optic_Nrv_PRV_L** | 0.842 | 0.836 | -0.7 | |
| **Optic_Nrv_PRV_R** | 0.81 | 0.804 | -0.7 | |
| **Optic_Nrv_R** | 0.68 | 0.690 | 1.5 | |
| **Pituitary** | 0.228 | 0.225 | -1.3 | |
| **PTV60** | 0.897 | 0.897 | 0.0 | |
| **PTV60ex** | 0.884 | 0.885 | 0.1 | |
| **PTV75** | 0.87 | 0.870 | 0.0 | |
| **STRUCTURES** | **DICE COEFFICIENT: "07-XXX-DUMMY"** | | | |
| **ROI Name** | **GOLDEN RULE** | **PLASTIMATCH** | | **DIFFERENCE (%)** |
| **Brain** | 0.941 | 0.945 | | 0.4 |
| **Brain-GTV** | 0.941 | 0.962 | | 2.2 |
| **Brainstem** | 0.888 | 0.886 | | -0.2 |
| **Brainstem_Centre** | 0.855 | 0.851 | | -0.5 |
| **Brainstem_PRV** | 0.897 | 0.895 | | -0.2 |
| **Cochlea_L** | 0.805 | 0.768 | | -4.7 |
| **Cochlea_R** | 0.763 | 0.774 | | 1.4 |
| **CTV60** | 0.927 | 0.927 | | 0.0 |
| **CTV60ex** | 0.929 | 0.928 | | -0.1 |
| **Eye_L** | 0.904 | 0.904 | | 0.0 |
| **Eye_R** | 0.872 | 0.869 | | -0.3 |
| **Glnd_Lacrimal_L** | 0.655 | 0.638 | | -2.6 |
| **Glnd_Lacrimal_R** | 0.626 | 0.642 | | 2.5 |
| **GTV** | 0.922 | 0.920 | | -0.2 |
| **GTVu** | 0.919 | 0.919 | | 0.0 |
| **Lens_R** | 0.822 | 0.800 | | -2.7 |
| **OpticChiasm** | 0.843 | 0.843 | | 0.0 |
| **OpticChiasm_PRV** | 0.911 | 0.901 | | -1.1 |
| **Optic_Nrv_L** | 0.756 | 0.752 | | -0.5 |
| **Optic_Nrv_PRV_L** | 0.805 | 0.803 | | -0.2 |
| **Optic_Nrv_PRV_R** | 0.81 | 0.809 | | -0.1 |
| **Optic_Nrv_R** | 0.722 | 0.715 | | -1.0 |
| **Pituitary** | 0.451 | 0.426 | | -5.7 |
| **PTV60** | 0.929 | 0.929 | | 0.0 |
| **PTV60ex** | 0.925 | 0.926 | | 0.1 |
| **PTV75** | 0.911 | 0.910 | | -0.1 |
